# Jaw Exercise Therapy and Psychoeducation to Reduce Oral Parafunctional Activities for the Management of Persistent Dentoalveolar Pain

**DOI:** 10.1155/2018/5042067

**Published:** 2018-09-12

**Authors:** Izumi Makino, Young-Chang Arai, Shuichi Aono, Masayuki Inoue, Hiroki Sakurai, Yusuke Ohmichi, Kazuhiro Shimo, Makoto Nishihara, Jun Sato, Noboru Hatakeyama, Takako Matsubara, Tatsunori Ikemoto, Takahiro Ushida

**Affiliations:** ^1^Multidisciplinary Pain Center, Aichi Medical University School of Medicine, Aichi, Japan; ^2^Institute of Physical Fitness, Sports Medicine and Rehabilitation, Aichi Medical University School of Medicine, Aichi, Japan; ^3^Faculty of Health and Medical Sciences, Tokoha University, Shizuoka, Japan; ^4^Rehabilitation, Nihon Fukushi University, Aichi, Japan

## Abstract

**Objective:**

To retrospectively analyze the effects of our original combination therapy treatment on patients with nonodontogenic persistent dentoalveolar pain.

**Methods:**

Twenty-one patients suffering from persistent dentoalveolar pain (nineteen females and two males; mean age ± standard deviation: 55.7 ± 19.6 years) participated in this study. They were treated with a therapy combination of jaw exercise and psychoeducation to reduce oral parafunctional activities every month. The intensity of pain in these subjects was evaluated using a numerical rating scale (NRS) before and after treatment.

**Results:**

The NRSs at the baseline ranged from 5 to 10 (median, 8), from 0 to 10 (median, 2) at one month after treatment, from 0 to 10 (median, 1) at three months after treatment, and from 0 to 10 (median, 0) at the end of treatment. Pain intensity after treatment improved significantly.

**Conclusion:**

There was a significant reduction in pain after our combination of therapies as nonpharmacological treatments, and therefore this treatment could be useful in the management of NPDP patients.

## 1. Introduction

Patients suffering from nonodontogenic persistent dentoalveolar pain (NPDP) continue to seek pain treatment from several doctors, not only dentists but also several kinds of medical practitioners. NPDP is known as idiopathic periodontalgia [1], phantom tooth pain [2], atypical odontalgia (AO) [3], persistent idiopathic facial pain [4], and persistent dentoalveolar pain disorder (PDAP) as recently reported by Nixdorf and Moana-Filho [5]. NPDP is described as a pain that persists in the dentoalveolar region without any evidence of clinical disease. Malacarne et al. reported PDAP is likely to be neuropathic in origin, but pathophysiological mechanisms to explain the onset and persistence of the pain are still far from understood in their review [6]. Dentists and medical practitioners treating NPDP frequently fail to provide adequate pain relief and improve patient's quality of life [7].

Several studies have shown that, in many cases of NPDP, the pain occurred after dental treatment, including endodontic treatment or surgical procedure [8, 9]. However, NPDP can also occur spontaneously in sound teeth [10]. Some reports suggested that NPDP was caused by central sensitization mechanisms [10–13]. Moreover, NPDP has been regarded as a psychological problem because the psychiatric prevalence of the PDAP patient was high since chronic pain disorder could be associated with psychological problems [1, 2, 5, 14]. Otherwise, NPDP might occur from cracked teeth or from myofascial pain syndrome [15], because it is difficult to differentiate the diagnoses.

Effective treatments for NPDP have yet to be established. General treatments for NPDP are the pharmacological treatments used to treat neuropathic pain. However, these treatments are not actually always effective in alleviating NPDP, and some patients experience severe side effects [7, 16]. Furthermore, several reports highly recommend avoiding irreversible treatments for NPDP [5, 8]. The current treatment target might be to improve quality of life (QOL) but not to provide adequate pain relief [6].

Recently, there are many reports that therapies including exercise therapy, cognitive-behavioral therapy or progressive relaxation, and intensive interdisciplinary rehabilitation are moderately effective for chronic pain. Therefore, we tried to treat NPDP patients using our original combination therapy (exercise of jaw movement and psychoeducation to reduce oral parafunction activities (OPAs)) that was effective on craniocervical chronic pain in a previous study [17].

The aim of this study was to retrospectively analyze the effects of treatment of our original combination therapy for patients with NPDP.

## 2. Materials and Methods

Data were collected in a test battery using an iPad and through interviews conducted by a dentist. The same dentist treated the patients using our original combination therapy. The intensity of pain was rated by the patient using a Numerical Rating Scale (NRS), where 0 indicated no pain and 10 indicated the greatest pain possible. Patients were asked about the pain intensity at every visit, and inquiries about their pain condition were made by telephone call for the follow-up.

All patients were referred from other hospitals to the Pain Center for treatment of NPDP. Treatment protocols used in the present report were based on institutional policy and clinical guidelines approved by the IRB of Aichi Medical University, and written consent was obtained from all patients (Reference number 13-083).

### 2.1. Participants

Participants were the patients who visited the Multidisciplinary Pain Center (Pain Center) of Aichi Medical University from December 2010 to June 2012. Twenty-one patients were chosen as participants at first, but six patients dropped out during treatment. Nineteen patients were female and two were male, and their mean age was 55.7 ± 19.6 years. They were selected according to the following inclusion and exclusion criteria:

  Inclusion criteria:
  (i) Nonodontogenic persistent dentoalveolar pain (NPDP)
  (ii) Pain duration more than 3 months
  (iii) Age >18 years
  Exclusion criteria:
  (i) Clear case of odontogenic pain
  (ii) Rheumatic disease or general inflammatory condition
  (iii) Fibromyalgia
  (iv) Language difficulties and communication difficulties
  (v) Closed locking jaw

### 2.2. Procedure of Treatment

The patients were administered our combination therapy under the supervision of a dentist (Makino) at our pain center at every visit. They were instructed to continue the exercises at home.

#### 2.2.1. Listening and Explanation

After checking the medical history of each patient, we listened to the patient's complaints regarding NPDP and reconfirmed their pain. We confirmed that there was no clinical or radiographic evidence of relevant pathology in their pain and explained this to the patients.

#### 2.2.2. Exercise for Jaw Movement

**Figure 1:**
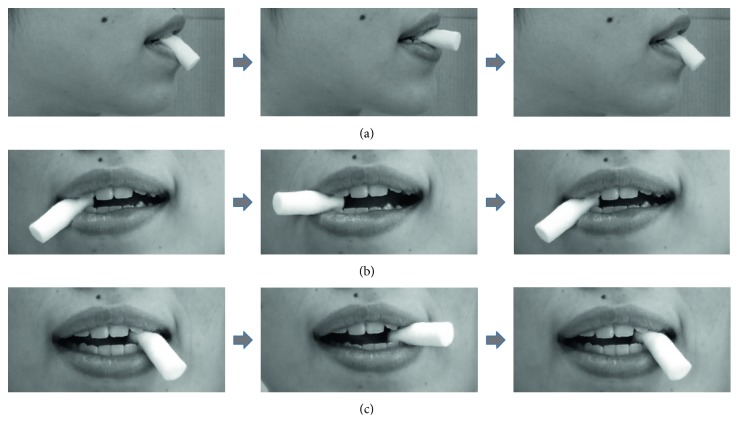
Jaw exercise. (a) Protusion-retrusion jaw movements while biting on a cotton roll with the front teeth. (b) Right jaw movement while biting on a cotton roll with the right canine. (c) Left jaw movement while biting on a cotton roll with the left canine.

Patients were instructed to perform the jaw movement exercise at home. The exercise consists of ten sets of protrusion-retrusion (anterior-posterior) jaw movements, a lateral jaw movement with the right side, and then a lateral jaw movement on the left side. Protrusion-retrusion (anterior-posterior) jaw movements were performed while biting on a cotton roll with the front teeth. A lateral jaw movement with the right side was performed while biting on a cotton roll with the right canine, and then a lateral jaw movement on the left side was performed while biting on a cotton roll with the left canine. One set exercise: a cotton roll goes up from below to the occlusal plane at first, secondly, a cotton roll maintains position at the occlusal plane, and finally, a cotton roll goes down in each exercise. These exercises were performed once a day. It was important for the patients to concentrate on the jaw exercise while watching their jaws move in a small mirror and to move the jaw slowly. They were also advised to perform the exercise carefully so as not to induce pain (Figure 1).

#### 2.2.3. Psychoeducation

*(1) Recognition of OPAs*. We informed patients to understand that it was important to recognize both OPAs and the frequency of OPAs during the daytime. We advised them not to be obsessed with OPAs or attempt to avoid them, but just to recognize that it had occurred. In addition, our other instruction was that when they felt pain, they had to recognize what kind of OPAs they were doing and how frequently they were doing them.

**Figure 2:**
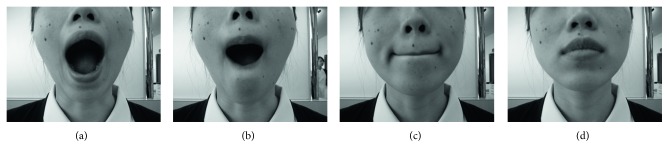
Mouth exercise and relaxation. (a) Take a breath with the mouth wide open. (b) Roll the lips back inside the mouth. (c) Close the mouth until the lips touch. (d) Relax the masticatory muscles, tongue, and jaw without letting the teeth touch.

*(2) Relaxation of Masticatory System Method*. Patients were trained to relax their tongue, their masticatory muscles, and their jaw. This form of relaxation involved taking a breath with their mouth wide open and then closing the mouth and trying to relax the tongue and jaw, without allowing the teeth to clench together. They were instructed to perform this system of relaxation when they noticed their pain and OPAs (Figure 2).

### 2.3. One-Year Follow-Up

In the follow-up 1 year after the patient's last visit, we followed up on their pain condition over a telephone call.

### 2.4. Statistical Analysis

The pain intensities in the patients gained at every visit to the pain center. The data of the initial pain and the pain intensity one month later, three months later, and at the end of treatment were first analyzed by Friedman test followed by Wilcoxon signed-rank test between the initial pain and the pain intensity one month later, three months later, and at the end of treatment. The level of significance for each test was set at *P*=0.01.

## 3. Results

The characteristics and the dental treatment histories of the patients are described in Table 1. The mean duration of pain was 37.7 ± 48.6 months. At first, 21 participants were recruited for this study, but six participants (patient number: 16-21) dropped out during treatment. Finally the number for statistical analysis was 15 out of 21 participants. Only 12 participants could be contacted by telephone call for the follow-up.

### 3.1. Intensity of Pain

**Figure 3:**
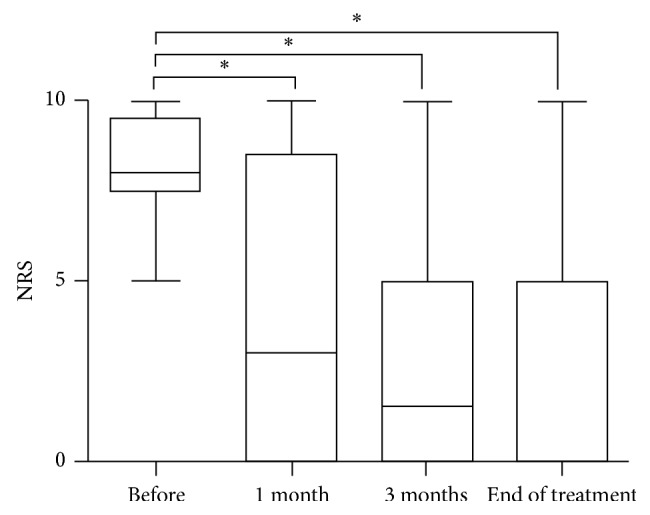
The pain intensity in each visiting time at pain center. Horizontal bar represents medians, boxes represent the 25th and 75th percentile ranges, and T bars represent the 5th and 95th percentile ranges. ^*∗*^Difference between before and after treatment (*P* < 0.01) (Wilcoxon test).

The individual changes in NRS are listed in Table 2. The NRSs of the baseline ranged from 5 to 10 (Median, 8). The NRS ranged from 0 to 10 (Median, 0) at one month after treatment, from 0 to 10 (Median, 0) at three months after treatment, and from 0 to 10 (Median, 0) at the end of treatment. The data of the 4 groups were significantly different by the Friedman test (*P* < 0.01). Pain intensity after treatment improved significantly (*P* < 0.01) (Wilcoxon test) (Table 2; Figure 3).

### 3.2. Follow-Up

One year later, we evaluated 11 patients by telephone call after the patient's last visit. Three patients out of 11 had no pain, seven patients had a recurrence of less pain, and one patient displayed no improvement. Ten patients took no medication, while one patient occasionally took medication. However, one patient took medication everyday with no improvement of pain (Table 3).

## 4. Discussion

Generally, patients with NPDP are suffering from their pain for a long time because available treatments fail to provide adequate pain relief. However, this study found that our treatment significantly improved the pain intensity of NPDP and their QOL at follow-up. To date, therapeutic algorithm for NPDP [8] includes mainly pharmacological treatments, which are tricyclic antidepressants [14], serotonin and norepinephrine reuptake inhibitors [18], anticonvulsants [19], or pregabaline [20] as used for neuropathic pain. Although these treatments could have serious side effects, the effectiveness is not so high for NPDP [16]. In our study, three patients said that they felt a little decrease in pain intensity after taking anxiolytics, but almost no patients experienced any pain relief through pharmacological treatments. These NPDP patients might include patients with persistent periodontitis without bacterial inflammation, myofascial pain syndrome, neuropathic pain, and PDAP because diagnostic criteria have not yet been established. But, the present results apparently showed the benefits of our original therapy for NPDP patients.

Our original therapy consists of exercise and psychoeducation that have become common therapies for chronic pain. Recently, there are many reports of moderately effective nonpharmacologic therapies for TMD [21, 22], and nonspecific chronic low back pain includes exercise therapy, cognitive-behavioral therapy or progressive relaxation, and intensive interdisciplinary rehabilitation [23]. Moreover, we have been focusing on the fact that many NPDP patients performed OPAs such as tongue touching of teeth and area of pain, and teeth clenching while experiencing pain or not. Many studies reported that chronic pain disorders including NPDP have been linked to psychological factors [5, 24]. And, a study reported that OPAs are associated with emotional stress induced by social factors [25]. Vickers et al. hypotheses that activation of low-threshold mechanoreceptors by bruxism, which is another form of OPA, may modulate nociception through gate-control mechanisms; bruxism may thus be a potential pain-coping mechanism for AO [24]. In this study, almost all patients claimed that they were always doing OPAs to pay attention to their pain area during the day. We announce that NPDP patients have been doing OPAs more frequently than dentists recognize, and OPAs keep stimulating teeth or pain area by hard pressure. That is, OPAs (teeth clenching or touching teeth by tongue) may be major triggers in NPDP, and highly frequent OPAs might have a bad influence on masticatory muscles. We think that psychoeducation to reduce the frequency of OPAs could decrease OPAs and change the facilitating muscles with exercise. An additionally important point is that we, as medical professionals, must sufficiently recognize the patients' pain and explain it to them, because of the importance of the relation between the patient and the clinician [21].

The Hospital Anxiety (median, 11) and Depression Scale (median, 10) and Pain Catastrophizing Scale (Median, 40) of the participants in this study showed high score tendencies. Four out of the 6 subjects who dropped out in this study had mental diseases (bipolar disorder, schizophrenia, and obsessive-compulsive disorder). Medical practitioners may need to be careful of mental conditions or mental diseases of NPDP patients.

Based on the present results, we could generate a hypothesis about the onset mechanisms of NPDP. NPDP patients are iterating OPAs (clenching and tongue habit) pressure teeth or area where a tooth once resists. If these pressures continue, c-fibers on periodontal ligament space will keep receiving the stimulation at first, and wind-up phenomena will occur subsequently at the central nervous system. This could be why local anesthesia for NPDP patients leads to complete or incomplete pain relief, as seen on a previous study [11]. And, most NPDP patients in this study said that they have more OPAs than they had thought, and their pain intensity became lower the second time they visited the pain center after they had controlled the OPAs. It seemed that the NPDP improved as the frequency of OPAs decreased. As NPDP is chronic pain that means a plastic change in the central nervous system, the pain could not be relieved immediately and psychoeducation is needed for the treatment. That is, in NPDP, inflammation on periodontium is processed under disrupted psychosocial conditions, thereby leading to plastic changes of the central nervous system.

There are several limitations to this study. First of all, the participants in this study were an undefined group having NPDP because correct diagnosis and pathophysiological mechanisms of NPDP have yet to be established. The number of participants in this study is small. The further studies of NPDP are needed but these studies are difficult because the population of NPDP patients is small.

## 5. Conclusion

This study reported a significant reduction in pain after our own combination of therapies to improve jaw movement and psychoeducation to reduce OPAs as forms of nonpharmacological treatment. Therefore, this treatment could be useful in the management of NPDP patients.

## Figures and Tables

**Table 1:** Characteristics and treatment used in 21 patients.

Patientnumber	Sex	Age (years)	Duration (months)	Pain location	History of present illness	Phamacological treatment	OPA	HADS		PCS
								(anxiety)	(depression)	
1	F	70	30	34	rct	NSAIDs	c, t	14	15	—
2	F	37	4	14.15	none	Tizanidine hydrochloride NSAIDs	c	6	5	36
3	F	58	7	(21) area	ext and cur	NSAIDs	t	7	5	23
4	F	27	7	16.26.36.46	rct	Mirtazapine-lorazepam	c, t	16	16	40
5	F	59	48	36.37	none	Flunitrazepam-etizolam	c, t	18	18	51
6	F	71	6	36.37	cr	Etizolam	c, t	9	3	40
7	F	39	48	26.27	none	Lorazepam	c	10	6	32
8	F	37	36	(26) area	ext and cur	Clonazepam-gabapentin carbamazepine-pregabalin	c, t	9	8	19
9	F	74	36	(46) area	rct and ext	NSAIDs	—	12	10	47
10	F	75	240	(24.25.26) area	ext	NSAIDs	c, t	8	4	42
11	F	82	24	11.14.21	none	NSAIDs	c	3	13	31
12	M	58	24	6.17.26.27.36.37.46.47	none	Pregabalin-brotizolam	j	13	14	37
13	M	78	24	37	br	NSAIDs	t	8	8	38
14	F	89	3	16 (44.45.46.47) area	ext and cur	Mirtazapine	t	15	18	50
15	F	72	3	11.12.13 (16) area	none	Pregabalin-carbamazepine NSAIDs	c, t	12	16	45
16	F	44	36	25 (26) area	ext	Drugs for bipolar disorder	c	18	18	—
17	F	39	30	26	rct and ext	Drugs for schizophrenia	c, t	7	11	22
18	F	46	36	13.38	none	Drugs for schizophrenia	c	11	18	44
19	F	69	36	(37.38) area	ext	Amitriptyline-NSAIDs-pregabalin	—	3	2	15
20	F	24	18	All teeth	none	Drugs for obsessive-compulsive disorder	—	13	9	46
21	F	21	96	26	None → pulpectomy	Amitriptyline	c	14	9	40
Mean		55.7	37.7					10.8	10.8	36.7

Tooth number is shown by two-digit notation (FDI). (Number) area is in situ after extraction of tooth. History of present illness: none = nontreatment; ext = extraction of tooth; rct = root canal treatment; cur = curettage of alveolar bone. cr = crown, br = bridge, OPA: c = diurnal tooth clenching; t = touching tooth by tongue; j = jaw moving; HADS = Hospital Anxiety and Depression Scale to determine the levels of anxiety and depression. PCS = Pain Catastrophizing Scale to determine the levels of physical and emotional distress associated with their pain.

**Table 2:** The pain intensity in each visiting time at pain center.

Patient number	Before	1 month	3 months	End
1	8	5	5	0
2	8	2	2	0
3	5	3	0	0
4	8	10	0	0
5	8	8	4	1
6	5	2	1	1
7	7	0	0	0
8	10	10	10	10
9	9	9	5	5
10	8	0	6	5
11	8	0	0	0
12	8	1	0	0
13	8	8	2	0
14	10	0	0	0
15	10	0	0	0
Mean	8	4	2	1
Median	8	2	1	0
*P* value		0.0051	0.007	0.007

The NRSs of the baseline ranged from 5 to 10 (median, 8). The NRS ranged from 0 to 10 (median, 0) at one month after treatment. The NRS ranged from 0 to 10 (median, 0) at three month after treatment. The NRS ranged from 0 to 10 (median, 0) at the end of treatment. *P* value: the data were analyzed by Wilcoxon signed-rank test between the initial pain and the pain intensity at one month later, three month later, and end of treatment.

**Table 3:** The condition in the follow-up.

Patient number	NRS	Medicine
1	3∼6	None
2	1∼2	None
3	0	None
4	2∼3	None
5	0	None
6	3∼5	Sometime
7	0	None
8	10	Usual
9	—	—
10	0∼1	None
11	3∼8	None
12	3∼5	None
13	0∼5	None
14	—	—
15	—	—

The NRS ranges from 0 to 10, and ten patients took no medication, one patient occasionally took medication in the follow-up.

## Data Availability

The data used to support the findings of this study are available from the corresponding author upon request.
